# Personality traits in patients with cluster headache: a comparison with migraine patients

**DOI:** 10.1186/s10194-016-0618-9

**Published:** 2016-03-15

**Authors:** I. Muñoz, M. S. Hernández, S. Santos, C. Jurado, L. Ruiz, E. Toribio, E. M. Sotelo, A. L. Guerrero, V. Molina, F. Uribe, M. L. Cuadrado

**Affiliations:** Psychiatry Department Hospital Clínico Universitario, Valladolid, Spain; Neurology Department, Hospital Clínico Universitario Lozano Blesa, Zaragoza, Spain; Neurology Department, Hospital Universitario Reina Sofía, Córdoba, Spain; Neurology Department, Hospital Universitario del Henares, Coslada, Madrid Spain; Neurology Department, Hospital Clínico Universitario, Avda. Ramón y Cajal 3, 47005 Valladolid, Spain; Neurology Department. Hospital Clínico San Carlos, Universidad Complutense, Madrid, Spain

**Keywords:** Cluster headache, Eccentric disorders, Migraine, Paranoid personality, Personality traits, *Salamanca* test, Schizoid personality

## Abstract

**Background:**

Cluster headache (CH) has been associated with certain personality traits and lifestyle features, but there are few studies assessing personality profiles in CH. We aimed to analyze personality traits in patients with CH, and to compare them with those found in migraine.

**Methods:**

We included all consecutive patients with CH attending 5 outpatient offices between January and December 2013. Personality traits were evaluated using the *Salamanca* screening test, a validated inventory assessing 11 personality traits grouped in 3 clusters. We analyzed the test results in this population, and compared them with those of a migraine population previously assessed with the same test.

**Results:**

Eighty patients with CH (75 men, 5 women; mean age, 43.2 ± 9.9 years) were recruited. The reference population consisted of 164 migraine patients (30 men, 134 women; mean age 36.4 ± 12.7 years). In CH patients, the most frequent personality traits were anancastic (52.5 %), anxious (47.5 %), histrionic (45 %), schizoid (42.5 %), impulsive (32.5 %) and paranoid (30 %). When compared to migraine patients, paranoid (*p* < 0.001; *χ*2 test), and schizoid traits (*p* = 0.007; *χ*2 test) were significantly more prevalent in CH patients. In logistic regression analysis the paranoid trait was significantly associated with CH (*p* = 0.001; OR: 3.27, 95 % CI [1.66–6.43]).

**Conclusion:**

According to the *Salamanca* screening test, personality traits included in cluster A (odd or eccentric disorders) are more prevalent in CH patients than in a population of migraineurs. Larger studies are needed to determine whether certain personality traits are related to CH.

## Background

Trigeminal autonomic cephalalgias (TAC) are a group of primary headache disorders that share the clinical features of unilateral headache, and often prominent cranial autonomic features ipsilateral to the headache [1]. Cluster headache (CH) is the most common form of TAC and one of the most severe forms of primary headache [2–4]. According to the International Classification of Headache Disorders, third edition, beta version (ICHD-3, beta) [5], CH is characterized by a severe or very severe unilateral head pain lasting from 15 to 180 min and occurring from once every other day to eight times a day, accompanied by ipsilateral autonomic symptoms and/or restlessness or agitation. CH attacks commonly have a striking circadian rhythm, with the attacks occurring at the same time each day, often in the afternoon or evening and during the night. In addition, episodic CH tends to have a circannual rhythm, with the active periods recurring at the same time of the year. A minority of patients have chronic CH, with the attacks occurring for more than one year without remission, or with remissions lasting less than one month.

Most patients with CH are men (3:1). CH leads to significant consequences on patients’ professional and social relationships and quality of life, along with a high use of health-care resources [6]. Psychiatric comorbidity in migraine is common and is increasingly being well documented, especially as it relates to major depressive disorder and the spectrum of anxiety disorders [7]. Contrarily to migraine, psychiatric comorbidity of CH patients has not been broadly assessed apart from few case series [8]. CH has been associated with anxiety disorders [9–11] and, mainly, depression [12]. In a large chronic cluster population from France, Donnet et al. noticed depression in 43 % [13]. CH has been nicknamed the suicide headache. Rozen et al. looked at the rate of suicidal ideations or true suicide attempts in a large CH population in USA. They found that an alarming 55 % of the survey responders stated they have had thoughts about suicide, and a 2 % have actually tried to commit suicide [14].

Personality traits might affect disability, resource utilization or adherence to therapies in headache patients. Previous studies have reported that levels of emotional functioning and perception of pain, independent of level of pain at baseline, were predictive of frequency, intensity, and duration of headaches [15]. Patients with headache and premorbid personality with important neurotic characteristics, understood as a personality with significant emotional instability and high levels of anxiety and worry, often have a bad pain management. This might affect the effectiveness of the usual therapies and therefore influence the prognosis of headache, for example with a transformation to a chronic form. The obsessive-compulsive personality trait has been the most frequently related [16, 17].

We aimed at analyzing personality traits in a population of CH patients using a validated screening test, and to compare them with those found in a migraine population.

## Methods

We assessed all consecutive patients with CH attending five headache outpatient offices from January 2013 to December 2013. CH was diagnosed according to both the second and third editions of the ICHD criteria [5, 18]. We obtained a complete history from each patient recording the demographic and clinical data systematically. We excluded patients with other headache diagnoses (including migraine), as well as patients with a medical or psychiatric disorder, not included within mood disorders or anxiety, or intellectual deficit that would limit their ability to understand or answer the questions of the questionnaires are included. The evaluation of depressive and anxiety symptoms was performed with the Hospital Anxiety and Depression Scale developed by Zigmond and Snaith [19]. It is a self-assessment scale, consisting on 14 items grouped into two subscales: anxiety and depression. The cutoff established for each of the subscales is 10. The HADS is especially useful for headache sufferers, since none of the 14 items refers to somatic symptoms, eliminating the risk of an overestimation of psychopathology derived from the interpretation of physical symptoms associated with headache secondary to anxiety or depression.

The study was approved by the Ethics Committee of the University Hospital of Valladolid, and all patients gave informed consent before participation.

We evaluated personality traits using the original Spanish version of the *Salamanca* screening test, a short, validated, and easy-to-interpret test, suitable for use in a neurology outpatient office [20]. The Salamanca test was designed to screen the eleven specific personality traits by using a total of 22 questions, two for each trait. There are four answers for each question. To screen the traits, for each answer a maximum of three points can be given. Thus, for each trait a scale from 0 to 6 indicates the degree of matching that specific personality trait. One of the main qualities of this test is its high sensitivity which leads to an early detection of personality features which can be sufficiently subtle so as not to be readily evident. Table 1 shows the English certified translation of the *Salamanca* test.

**Table 1:** English translation of Salamanca Test

1. I am reluctant to confide in others	T	sometimes	frequently	always	F
		1	2	3	
2. I would like to give others what they deserve	T	sometimes	frequently	always	F
		1	2	3	
3. I prefer solitary activities	T	sometimes	frequently	always	F
		1	2	3	
4. I prefer being alone	T	sometimes	frequently	always	F
		1	2	3	
5. Do people think you are strange or eccentric?	T	sometimes	frequently	always	F
		1	2	3	
6. I am more in touch with the paranormal than most people	T	sometimes	frequently	always	F
		1	2	3	
7. I am too emotional	T	sometimes	frequently	always	F
		1	2	3	
8. I care a lot about my image	T	sometimes	frequently	always	F
		1	2	3	
9. I do illegal things	T	sometimes	frequently	always	F
		1	2	3	
10. I have little respect for the rights of others	T	sometimes	frequently	always	F
		1	2	3	
11. I am special and deserve to be recognized for it	T	sometimes	frequently	always	F
		1	2	3	
12. Many people are envious of me.	T	sometimes	frequently	always	F
		1	2	3	
13. My emotions are like a roller coaster	T	sometimes	frequently	always	F
		1	2	3	
14. I am impulsive	T	sometimes	frequently	always	F
		1	2	3	
15. I frequently wonder what my purpose in life is	T	sometimes	frequently	always	F
		1	2	3	
16. I am easily bored or have feelings of emptiness	T	sometimes	frequently	always	F
		1	2	3	
17. Do people think you are too perfectionist, obstinate or rigid?	T	sometimes	frequently	always	F
		1	2	3	
18. I am meticulous, thorough and too much of a hard-worker.	T	sometimes	frequently	always	F
		1	2	3	
19. I need to feel cared for and protected.	T	sometimes	frequently	always	F
		1	2	3	
20. I find it hard to make my own decisions.	T	sometimes	frequently	always	F
		1	2	3	
21. I am a nervous person.	T	sometimes	frequently	always	F
		1	2	3	
22. I am very afraid of being ridiculous.	T	sometimes	frequently	always	F
		1	2	3	

Personality traits assessed by the *Salamanca* test were grouped into three clusters as shown in Table 2. Cluster A included paranoid, schizoid and schizotypal traits; Cluster B comprised antisocial, narcissistic, histrionic, and emotional instability disorders subtypes limit and impulsive, while Cluster C included dependent, anancastic and anxious traits [21]. This test was previously administered to migraine patients attending the same outpatient clinics and coming from the same geographical areas [22].

**Table 2:** Personality traits assessed by Salamanca Test grouped into 3 Clusters

Cluster A	PAR	Paranoid (items 1 and 2)
	SCHZD	Schizoid (items 3 and 4)
	SCHT	Schizotypal (items 5 and 6)
Cluster B	HIST	Histrionic (items 7 and 8)
	ANT	Antisocial (items 9 and 10)
	NAR	Narcissistic (items 11 and 12)
	EU IMP	Emotionally unstable disorders
		Impulsife type (items 13 and 14)
	EU LIM	Emotionally unstable disorders
		Borderline type (items 15 and 16)
Cluster C	ANAN	Anancastic personality disorder. (items 17 and 18)
	DEP	Dependent (items 19 and 20)
	ANX	Anxious personality disorder (items 21 and 22)

Statistical analysis was conducted with the SPSS statistical package (version 20.0 Inc., Chicago, IL, USA). Univariate association between personality traits and type of headache (CH or migraine) was assessed with the *χ*2 test. Personality traits associated with any type of headache (*p* < 0.2 in univariate analysis) were subsequently included in a logistic regression analysis. As gender distribution was markedly different between CH and migraine patients, in order to exclude any influence of gender on personality traits, gender was included in a second regression analysis in each of the populations (CH or migraine). All models were constructed using backward and forward stepwise variable selection. A *p*-value of <0.05 was considered significant.

## Results

We included 80 patients with CH (5 female, 75 male). Mean age at inclusion was 43.2 ± 9.9 years (range: 23–64) and mean age at onset was 31.3 ± 11.8 (range: 10–57). At the time of assessment, 67 patients (83.8 %) had episodic headache, and 13 patients (16.2 %) had chronic CH. In the migraine group there were 164 patients (134 female, 30 male), with mean age at inclusion of 36.4 ± 12.7 years (range: 10–78) and mean age at onset of 19.5 ± 9.2 (range: 5–50). As expected, most patients with CH were male, in contrast with female migraine patients.

In the CH group, the most frequent personality traits were anancastic (52.5 %), anxious (47.5 %), histrionic (45 %), schizoid (42.5 %), impulsive (32.5 %) and paranoid (30 %). Most patients shared several personality traits, especially those belonging to cluster A.

A comparison between the distribution of personality traits in CH patients and migraine patients is shown in Table 3. Only two of the 11 personality traits assessed (dependent and anxious) were more frequent in migraine patients.

**Table 3:** Comparison between personality traits in Cluster headache patients and migraine patients

Personality traits	Cluster headache patients (n: 80)	Migraine patients (n: 164)	*P value (*χ*2 test)
Paranoid (%)	30 %	11.6 %	<0.001
Schizoid (%)	42.5 %	25.6 %	0.007
Schizotypal (%)	10 %	5.5 %	0.19
Histrionic (%)	45 %	40.9 %	0.53
Antisocial (%)	2.5 %	0 %	0.10
Narcisistic (%)	6.3 %	4.9 %	0.65
EIDSI (%)	32.5 %	23.8 %	0.14
EIDSL (%)	18.8 %	15.9 %	0.57
Anancastic (%)	52.5 %	44.5 %	0.24
Dependent (%)	27.5 %	32.9 %	0.39
Anxious (%)	47.5 %	53.7 %	0.36

*EIDSI* Emotional instability disorder subtype impulsive

*EIDSL* Emotional instability disorder subtype limit

In the first logistic regression analysis only the paranoid trait was significantly associated with CH (*p* = 0.001; OR: 3.27, 95 % CI [1.66–6.43]). No relationship between gender and personality traits was found in the second regression analysis within the CH or the migraine groups. Patients with episodic and chronic CH did not show different personality profiles (data not shown).

## Discussion

Patients with CH seem to be more vulnerable to diverse psychiatric comorbidities [8]. Depression is the best acknowledged psychiatric comorbidity in this headache; a recent study concluded that CH patients were 5.6 times more likely to develop depression than the control population [23]. Several authors have also tried to describe the mental profile of these patients [24–26]. A particular personality profile has been proposed for CH patients, which would be characterized by ambition, dependency and an inability to reveal their feelings [27]. They were even described as “mice living inside lions” [28]. Personality studies have shown that these patients are more prone to anxiety, hypochondriasis and hysteria [24, 29–31]. CH patients are less successfully socially integrated, and have a more hostile attitude towards others [24]. However, some authors maintained that data used for defining personality profiles just reflected coping resources of patients against pain [32]. Anyway, it has not been possible to define rigorously a personality profile for CH patients [26, 30, 33–35].

In contrast, relationships between migraine and psychiatric comorbidities have been more extensively analyzed [36–38], especially regarding the axis I of the Diagnostic and Statistical Manual of Mental Disorders, 4th Edition, Text Revision (DSM-IV-TR), [39, 40]. Concerning personality traits, axis II of DSM-IV-TR, this difference is even more remarkable [18, 41].

A personality trait is a stable and pervasive pattern of interpreting, perceiving, thinking about, and relating to one’s environment, as well as to oneself. It is stable over time. A personality trait affects individual’s life and cannot be explained by substance abuse, other mental disorder or medical conditions. The personality traits are grouped into three clusters: A, B and C. Cluster A comprises odd or eccentric traits, cluster B dramatic or emotional disorders traits and cluster C consists of anxious or fearful traits.

According to our results obtained using the *Salamanca* screening test, it seems that some personality traits belonging to Cluster A are quite common among CH patients, while personality traits included in Cluster C are apparently more frequent among migraine patients. Cluster C reflects an anxious and stress reactive personality while cluster A reveals a rigid way of thinking and feeling relating to the others. This means that most of the patients diagnosed with CH exhibit a paranoid-schizoid position. This position may influence their response to pain and result in an inadequate coping strategy. These results are similar to those obtained by other authors with the Minnesota Multiphasic Personality Inventory (MMPI) questionnaire, in which high paranoia and hypochondria levels ocurred among CH patients [42], but differed from others with comparable MMPI scores in CH and migraine patients [32, 43–45]. Otherwise, this study provides some evidence that psychiatric comorbidity may be related with primary headache itself, as already pointed out by Robbins [7].

Different personality profiles might be explained as a response to continued exposure to pain, as it would occur in chronic forms, or to the risk of sudden intense pain, as would happen in episodic pains [46]. We have not found different profiles in chronic or episodic CH, though the number of chronic forms in our series is too small to reach a conclusion. However headache chronification has been related with psychiatric comorbidity, due the significant role it might play in the way of perceive pain, cope with it and maintain a normal lifestyle. We could expect high scores in personality traits of chronic CH in a bigger series.

There are several major limitations of this survey study, the main one being that we do not have a matched healthy control population. As gender distributions between CH and migraine populations are quite different, they might mask any comparison concerning other variables. Yet, we have not found a significant influence of gender on personality traits in each population. Another problem was that the use of the *Salamanca* screening test helped us to evaluate personality traits in a neurology outpatient office but is considered a somewhat overinclusive instrument in “making diagnosis” [20]. Finally, the cross-sectional design of the study does not permit to reveal any casual relationship. Larger studies are needed to solve these limitations and to confirm whether personality traits are directly related to CH and if they differ from those related to other primary headaches.

## Conclusion

According to our results, personality pattern is different among migraine and Cluster Headache Patients. So, psychological profile may somehow be marked by the primary headache. This study would reflect a paranoid-schizoid personality among CH patients that might influence the response of these patients to therapy, or even their therapeutic alliance with neurologists. The personality pattern showed in our data could be related to a difficult coping with changes and pain.

## Consent

Consent to publish has been obtained from the participants.

## References

[1] Costa A, Antonaci F, Ramusino MC, Nappi C (2015). The neuropharmacology of Cluster Headache and other Trigeminal Autonomic Cephalalgias. Curr Neuropharmacol.

[2] Fischera M, Marziniak M, Gralow I, Evers S (2008). The incidence and prevalence of cluster headache: A meta-analysis of population-based studies. Cephalalgia.

[3] Larner AJ (2008). Trigeminal Autonomic cephalalgias: Frequency in a general neurology clinic setting. J Headache Pain.

[4] Jensen R, Lyngberg A, Jensen R (2007). Burden of cluster headache. Cephalalgia.

[5] Headache Classification Subcommittee of the International Headache Society (2013). The international classification of headache disorders: 3rd edition (beta version). Cephalalgia.

[6] D’Amico D, Rigamonti A, Solari A (2002). Health-related quality of life in patients with cluster headache during active periods. Cephalalgia.

[7] Jelinski SE, Magnusson JE, Becker WJ (2007). Factors associated with depression in patients referred to headache specialists. Neurology.

[8] Gesztelyi G, Bereczki D (2005). Disability is the major determinant of the severity of depressive symptoms in primary headaches but not in low back pain. Cephalalgia.

[9] Robbins MS, Bronheim R, Lipton RB (2012). Depression and anxiety in episodic and chronic cluster headache: a pilot study. Headache.

[10] Jürgens TP, Gaul C, Lindwurm A (2011). Impairment in episodic and chronic cluster headache. Cephalalgia.

[11] Jorge RE, Leston JE, Arndt S, Robinson RG (1999). Cluster headaches: Association with anxiety disorders and memory deficits. Neurology.

[12] Seifert CL, Valet M, Pfaffenrath V (2011). Neurometabolic correlates of depression and disability in episodic cluster headache. J Neurol.

[13] Donnet A, Lanteri-Minet M, Guegan-Massardier E (2007). Chronic cluster headache: A french clinical descriptive study. J Neurol Neurosurg Psychiatry.

[14] Rozen TD, Fishman BA (2012). Cluster headache in the United States of America: Demographics, Clinical Characteristics, Triggers, Suicidality, and Personal Burden. Headache.

[15] Pompili M, Gianluca S, Di Cosimo D (2010). Psychiatric comorbidity and suicide risk in patients with chronic migraine. Neuropsychiatr Dis Treat.

[16] Curone M, D’Amico D, Bussone G (2012). Obsessive-compulsive aspects as predictors of poor response to treatments in patients with chronic migraine and medication overuse. Neurol Sci.

[17] Bigal ME, Sheftell FD, Rapaport AM, Tepper SJ, Weeks R, Baskin SM (2003). MMPI personality profiles in patients with primary chronic headache: A case–control study. Neurol Sci.

[18] Headache Classification subcommittee of the International Headache Society (2004). The international classification of headache disorders second edition. Cephalalgia.

[19] Zigmond AS, Snaith RP (1983). The hospital anxiety and depression scale. Acta Psychiatr Scand.

[20] Caldero-Alonso A (2009). Estudio de los resultados obtenidos en el Cuestionario Salamanca en población normal.

[21] López-Ibor Aliño JJ, Valdés-Miyar M (2002). Manual diagnóstico y estadístico de los trastornos mentales.

[22] Muñoz I, Toribio-Diaz ME, Carod-Artal FJ (2013). Personality traits in patients with migraine: a multi-centre study using the Salamanca screening questionnaire. Rev Neurol.

[23] Liang JF, Chen YT, Fuh JL, Li SY, Liu CJ, Chen TJ (2013). Cluster headache is associated with an increased risk of depression: A nationwide population-based cohort study. Cephalalgia.

[24] Kudrow L (1974). Physical and personality characteristics in cluster headache. Headache.

[25] Italian Cooperative Study Group on the Epidemiology of Cluster Headache (ICECH) (2000). Case–control study on the edpidemiology of cluster headache: anthropometric data and personality profile. Funct Neurol.

[26] Levi R, Edman GV, Ekbom K, Waldenlind E (2005). Episodic cluster headache I: personality and some neuropsychological characteristics in male patients. Headache J Head Face Pain.

[27] Graham JR, Saxena PR (1975). Some clinical and theoretical aspects of cluster headache. Migraine and related Headaches.

[28] Graham JR (1972). Cluster headache. Headache.

[29] Rogado AZ, Harrison RH, Graham JR (1974). Personality profiles in cluster headache, migraine and normal controls. Arch Neurobiol.

[30] Evers S (2005). Cognitive processing in cluster headache. Curr Pain Headache Rep..

[31] Steinhilber RM, Pearson JS, Rushton JG (1960). Some psychological considerations of histaminic cephalalgia. Mayo Clin Proc.

[32] Robinson ME, Geisser ME, Dieter JN, Swerdlow B (1991). The relationship between MMPI cluster membership and diagnostic category in headache patients. Headache.

[33] Pfaffenrath V, Hummelsberg J, Pöllmann W, Kaube H, Rath M (1991). MMPI personality profiles in patients with primary headache syndromes. Cephalalgia.

[34] Mathew NT (1992). Cluster headache. Neurology.

[35] Zwart JA, Ellertsen B, Bovim G (1996). Psychosocial factors and MMPI-2 patterns in migraine, cluster headache. New Trends Exp Clin Psychiatry.

[36] Jette N, Patten S, Williams J, Becker W, Wiebe S (2008). Comorbidity of Migraine and Psychiatric Disorders. A National Population-Based Study. Headache.

[37] Hamelsky SW, Lipton RB (2006). Psychiatric comorbidity of migraine. Headache.

[38] Antonaci F, Nappi G, Galli F, Manzoni GC, Calabresi P, Costa A (2011). Migraine and psychiatric comorbidity: a review of clinical findings. J Head Pain.

[39] Lake III AE, Rains JC, Penzien DB, Lipchick GL (2005). Headache and psichiatric comorbidity: Historical context, clinical implications, and research relevance. Headache.

[40] Radat F, Swendsen J (2004). Psychiatric comorbidity in migraine: a review. Cephalalgia.

[41] Luconi R, Bartolina M, Taffi R, Vignin A, Mazzanti L, Provinciali L (2007). Prognostic Signifiance of personality profiles in patients with chronic migraine. Headache.

[42] Cupini LM, De Murtas M, Costa C, Mancini M, Eusebi P, Sarchielli P (2009). Obsessive-compulsive disorder and migraine with medication-overuse headache. Headache.

[43] Kudrow LO, Suktus BJ (1979). MMPI pattern specificity in primary headache disorders. Headache.

[44] Cuypers J, Altenkirch H, Bunge S (1981). Personality profiles in cluster headache and migraine. Headache.

[45] Watson D (1982). Neurotic tendencies among chronic pain patients: an MMPI item analysis. Pain.

[46] De Domini P, Del Bene E, Gori-Savellini S, Manzoni GC, Martucci N, Nappi G (1983). Personality patterns of headache sufferers. Cephalalgia.

